# Biological safety of Electroacupuncture with STS316 needles

**DOI:** 10.1186/s12906-019-2674-6

**Published:** 2019-10-28

**Authors:** Kwang-Ho Choi, Sun Hee Yeon, Seong Jin Cho, O Sang Kwon, Sanghun Lee, Su Yeon Seo, Suk-Yun Kang, Yeonhee Ryu

**Affiliations:** 1 Clinical Medicine Division, Korean Institute of Oriental Medicine, 305-811 Daejeon, Republic of Korea; 2 Department of Otorhinolaryngology-Head and Neck Surgery, Research Institute for Medical Science, Chungnam National University School of Medicine, Daejeon, South Korea; 3 College of Korean Medicine, Wonkwang University, Iksan, Republic of Korea

**Keywords:** Electroacupuncture, STS304, STS316, Corrosion, Necrosis, Cytotoxicity

## Abstract

**Background:**

Electroacupuncture (EA) is often used in clinical settings due to its analgesic effect, but its safety has not been verified due to the lack of clear criteria. This study examined the critical range of the corrosion of stainless steel types STS304 and STS316, which have been used clinically, and the relationship between needle corrosion and cell necrosis.

**Method:**

The critical point of corrosion for STS304 and STS316 was identified by varying the time, frequency, and stimulation intensity. In a tissue necrosis experiment, EA stimulation was applied to rats using STS316 needles with different thicknesses at maximum intensity for 60 min, and the presence of corrosion and tissue necrosis was determined. A cytotoxicity experiment was also conducted and assessed the needles and tissue necrosis.

**Results:**

The results showed that STS316 was more stable than STS304 and that only coated needles corroded. Furthermore, tissue necrosis was observed regardless of corrosion, and slight cell necrosis was associated with needles with corrosion.

**Conclusions:**

This study demonstrated that non-coated STS316 was the most stable for EA stimulation and that corrosion byproducts and cell necrosis were not directly related.

## Summary points

 ➔ The STS316 needle was more stable than the STS304 needle in terms of the degree of corrosion by electrical stimulation.

➔ Corrosion occurred only in the coated needle, and there was no direct correlation between the corrosion of needle and cellular necrosis by electroacupuncture (EA).

➔ Strong electrical stimulation on the needle may cause cytotoxicity regardless of corrosion.

## Introduction

Electroacupuncture (EA) is an electrical treatment used to alleviate and improve pain and other symptoms by delivering pulse-type currents to the body through a pair of needles placed on the area to be stimulated based on traditional medical theory. The identified mechanisms of EA treatment are analgesic action by central and peripheral nerve stimulation, including the pain control effect of opioids [1] and analgesic action by serotonin [2]. The alleviation of pain caused by musculoskeletal disorders, neurological disorders, childbirth, and stomach disorders [3] has been listed as the most effective use of EA in recent clinical studies.

Despite such a good therapeutic effect of EA stimulation, it is difficult to guarantee its biosafety because non-biological electrical stimulation is applied to the human body through stainless steel. Minerals or organic compounds may degrade stainless steel, while electrical stimulation can promote degradation [4]. The electrical corrosion of stainless steel may cause cytotoxicity due to corrosion byproducts and the leaching and accumulation of metal ions in the body [5, 6]. Thus, safety issues could be a concern depending on the conditions of EA, yet no clear criteria for the materials used to make acupuncture needles for EA or for the methods of EA operation have been suggested. Therefore, there is a need to conduct research on the safety of the materials in EA needles and of EA operation methods to improve the clinical safety of EA and to develop EA treatments that have no adverse side effects.

In preliminary studies on the evaluation of EA safety, Hwang et al. [7] tested the degree of corrosion caused by various intensities and lengths of EA stimulation and suggested that cell necrosis may be caused by corrosion byproducts. Lee et al. [8] compared corrosion among nine different needle materials under conditions of strong electrical stimulation within the range that can be used in clinics and concluded that STS316 is the most suitable material. However, additional experiments investigating the mechanism of cell necrosis caused by EA stimulation are required to provide guidelines on the safe use of EA.

Thus, this study aimed to experimentally confirm a safe range of conditions for the use of acupuncture needles composed of STS304 or STS316 based on the corrosion associated with current intensity, pulse frequency, and duration. Also, we aimed to determine the conditions associated with the corrosion of acupuncture needles during EA stimulation, such as the material, silicone coating, and acupuncture needle thickness. In addition, the presence of a silicone coating on STS316 needles of various thicknesses was investigated in terms of its effect on corrosion and tissue damage by EA, and the relationship between needle corrosion and cell necrosis was confirmed.

## Methods

### Safety of STS304 and STS316

#### Acupuncture

The experiment used needles composed of STS304 and STS316 that were coated with silicone for easier insertion into the human body (0.25 mm × 0.40 mm; disposable needle, DongBang Acupuncture, Korea). The needles were immersed in Hank’s solution (Table 1), and pulse-type voltage was applied.

**Table 1:** Contents of Hank’s solution

Component	Concentration (g/L)
NaCl	8.0063
KCl	0.4003
Na_2_HPO_4_	0.0483
KH_2_PO_4_	0.0572
CaCl_2_	0.1398
MgSO_4_	0.0641
NaHCO_3_	0.3503

#### Corrosion test

Electrical stimulation was performed using a low-frequency electrical stimulator (STN-111, Stratek, Korea) with an intensity ranging from 1 to 9. The power and duration according to intensity are shown in Table 2. Each frequency (1, 16, 30, and 60 Hz) was applied for 5, 10, 15, 60, or 90 min to measure corrosion based on intensity.

**Table 2:** Power and duration of pulses according to the intensity of low-frequency electrical stimulation

Intensity	1	2	3	4	5	6	7	8	9
Vpp (V)	0.3	20	70	150	220	250	340	350⤒	350⤒
Duration (μs)	40	75	80	80	80	80	80	80	80

### Tissue safety

#### Experimental animals

Sprague Dawley (SD) rats (12 weeks, 350 g) were purchased from Chungang Lab Animals (Seoul, Korea). The animals were kept for a week at 22 ± 1 °C with 55 ± 10% relative humidity and a 12-h light cycle, with 2–3 rats housed in a cage with soft bedding and free access to food and water. After the experiment, the animals were euthanized by cardiac injection of 10% urethane solution while they were still fully anesthetized.

#### EA stimulation of animals

Acupuncture point of ST32 (Bokto) and ST36 (Joksamni) was selected for the EA stimulation because the tissue has enough muscle and skin thickness for the test. Acupuncture points were identified on the body surface of the experimental animals corresponding to ST32 (Bokto) and ST36 (Joksamni) of the human body by bone proportional cun. The EA stimulation was conducted with STS316 needles (coated (silicone) or non-coated) with thicknesses of 0.18, 0.2, 0.25, or 0.3 mm and a frequency of 120 Hz with intensity of 9 (Table 2) for 60 min under mixed anesthesia of isoflurane (Ilsung Pharmaceuticals; Korea) and N_2_O/O_2_ gas. After 60 min of EA stimulation, the animals were sacrificed to collect the tissues from the area of EA stimulation.

#### Histological analysis

**Fig. 1:**
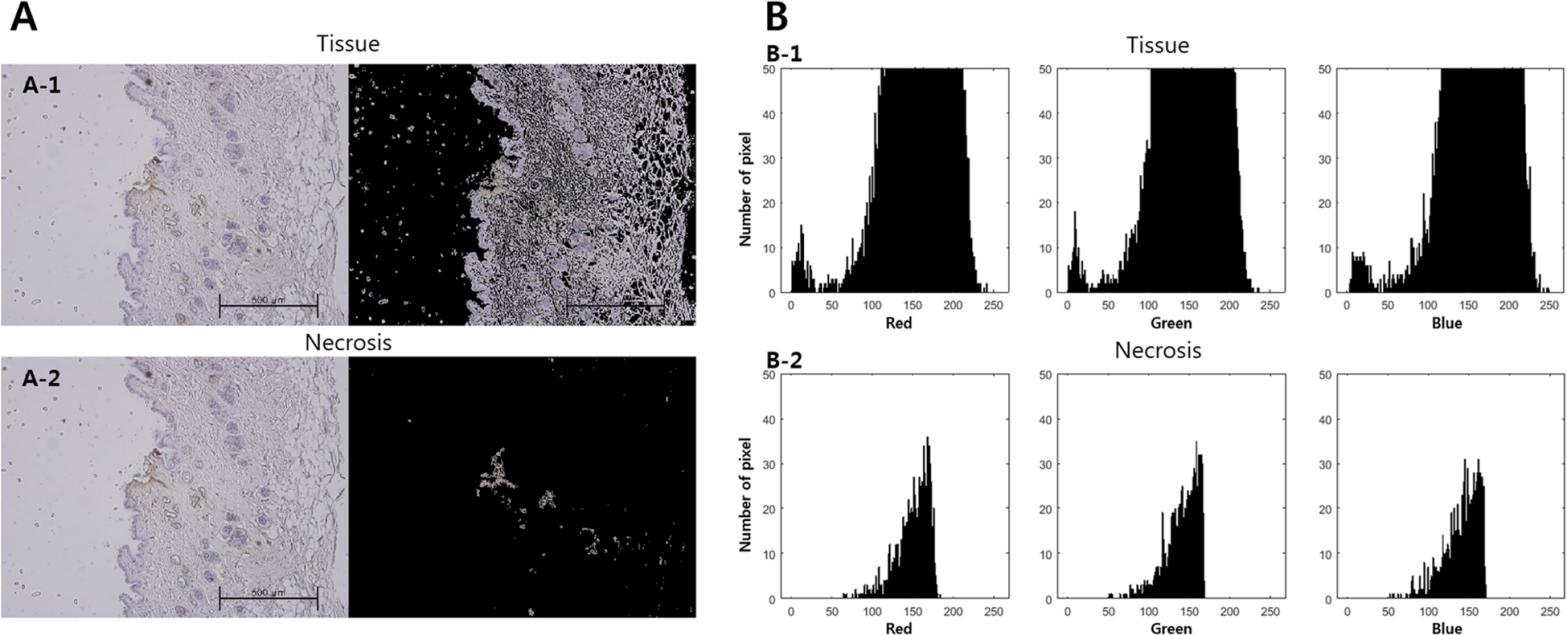
Quantitative analysis of tissues with necrosis. **a**. Photographs of tissues showing pixels (A-1) and tissue with necrosis showing pixels (A-2). **b**. Graphs showing RGB (red, blue, green) values and number of pixels of the whole tissue (B-1) and RGB values and number of pixels of the tissue with necrosis (B-2). The RGB values of the tissue with necrosis are shown in B-2, and the presence of tissue necrosis was determined by the number of pixels based on Fig. 1 (B-2)

**Fig. 2:**
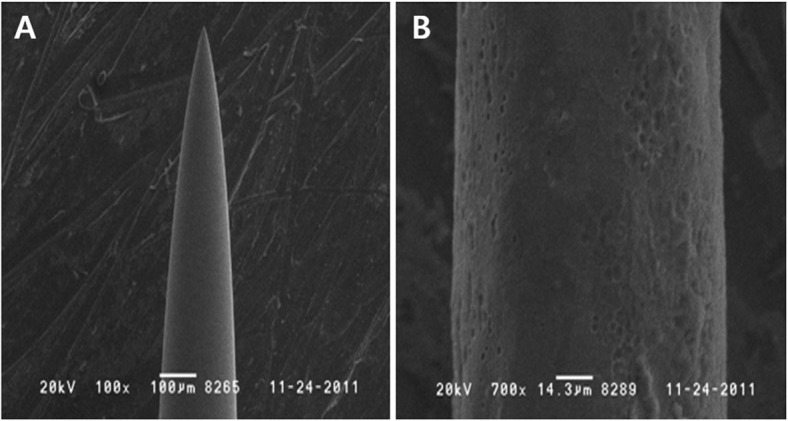
Presence of corrosion after applying electricity. **a**: Electron micrograph (× 100) of a needle without corrosion after applying electricity, **b**: Electron micrograph (× 700) of a needle with corrosion after applying electricity

The tissues were fixed in 10% neutral buffered formalin and embedded in paraffin, then sectioned using a microtome (LEICA RM2245, Germany). After deparaffinization and hydration, the terminal deoxynucleotidyltransferase-mediated dUTP nick end labeling (TUNEL) assay (ApopTag Plus Peroxidase In Situ Necrosis Detection Kit, S7101, Millipore, Darmstadt Germany) was used on the tissue slides. The TUNEL assay was performed according to the manufacturer’s instructions. After dehydration and clearing, samples were mounted using synthetic mountant (Shandon, USA) and observed under a light microscope (Dp70, Olympus, Japan). Quantitative analysis of tissue necrosis was performed using the image processing toolbox of the MATLAB (Mathworks, USA) program. As shown in Fig. 1 (B-2), the presence of tissue necrosis was determined by the number of pixels with RGB values.

### Cytotoxicity

#### Cells

The NCTC clone 929 (L-929) cell line, which is recommended by the International Organization for Standardization for cytotoxicity experiments, was obtained from the Korean Cell Line Bank. NCTC clone 929 (L-929) cells were incubated in RPMI 1640 media (GIBCO, NY, USA.) with 10% FBS and 1% penicillin/streptomycin at 37 °C and 5% CO_2_. NCTC clone 929 cells were counted (2 × 10^4^ cells/well), seeded onto a 96-well plate, and incubated at 37 °C for 24 h. 

#### Production of corrosion byproducts

The needles (non-coated 0.25 mm diameter STS316 needles and coated 0.2 and 0.3 mm diameter STS316 needles) which showed necrosis as a result of animal study described in the prior paragraphs were immersed in complete RPMI 1640 media (10% FBS, 1% p/s), and electrical stimulation was then applied at frequency of 120 Hz and intensity of 9 (Table 2) for 60 min with the low-frequency electrical stimulator. The samples were diluted to the complete RPMI 1640 media with a concentration of samples as 100, 50, 25, and 0% (control).

#### Cell viability

The RPMI media containing corrosion byproducts in the concentration described prior were applied just after the RPMI media had removed from the 96-well using a suction pump. The cells in the RPMI media containing corrosion byproducts were incubated for 48 h at 37 °C and 5% CO_2_.

After the incubation of 48 h, a total of 10 μl of 5 mg/ml MTT solution (Sigma, MO, USA) was added to each well, followed by incubation at 37 °C for 3 h. After crystals formed in the cells from the application of MTT solution, the media was gently removed with the suction pump, and 100 μl of 100% dimethyl sulfoxide (Sigma, MO, USA) was added to dissolve the crystals completely. Absorption was then measured at 570 nm using a ELISA microplate reader (MRX, Dynatech Laboratories, Chantilly, VA, USA).

Each experiment was repeated three times. The MTT assay results were compared using PASW Statistics 18 (Polar Engineering and Consulting, USA), and ANOVA with Tukey’s HSD post hoc test was performed, with significance set at *p* < 0.05.

### Observation of needle corrosion

The degree of corrosion of the STS304 and STS316 needles used in the experiments evaluating needle safety, tissue safety, and cytotoxicity was determined using a scanning electron microscope (Philips 525-M; Royal Philips Electronics, Inc., Netherlands) at × 200, × 300, × 500, × 1000, and × 5000 magnifications.

## Results

### Corrosion observed in the acupuncture needles

**Fig. 3:**
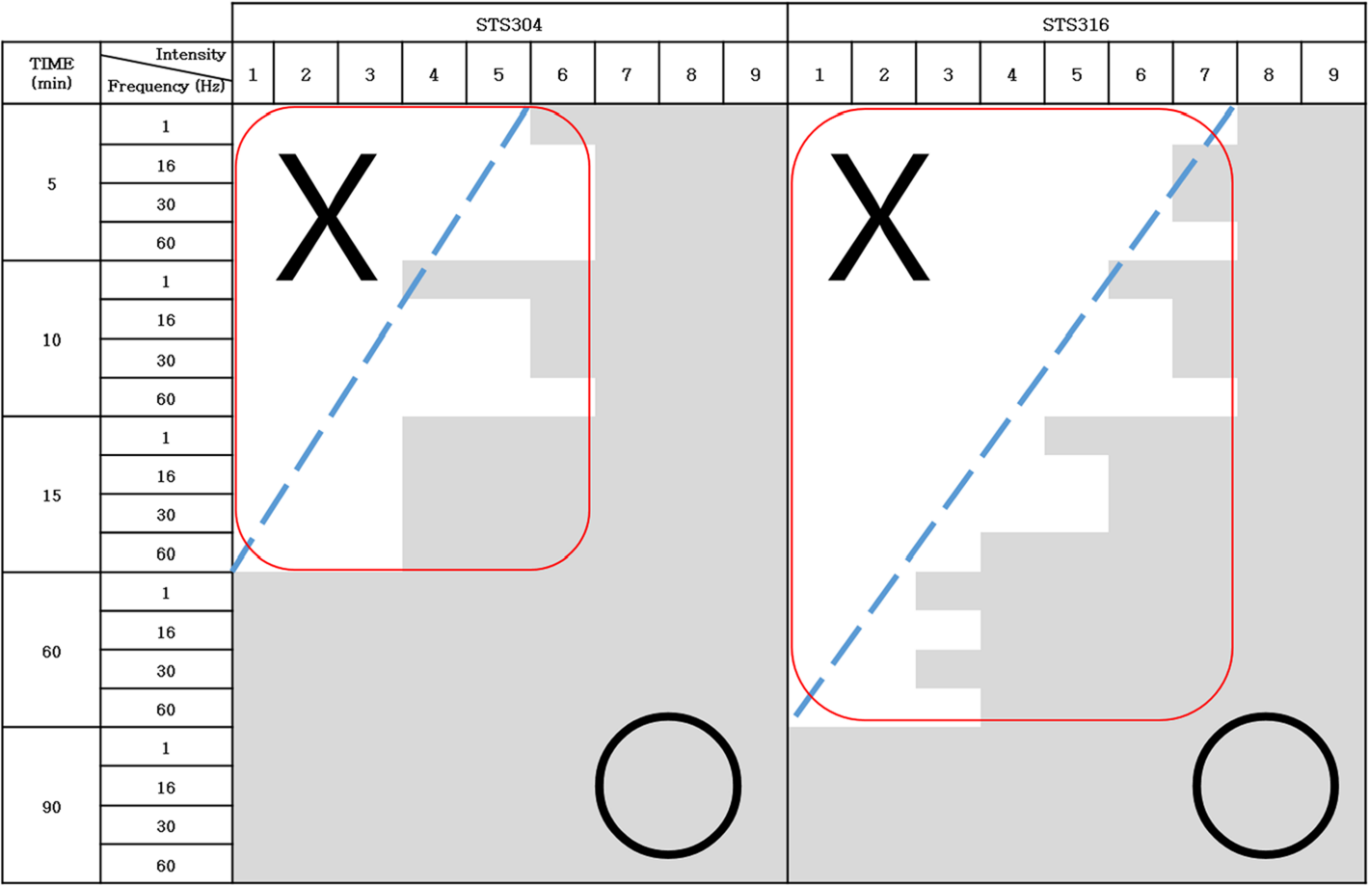
Comparison of corrosion in STS304 and STS316 according to electrical stimulation intensity, frequency, and time. The sections shaded gray indicate corrosion (○), and the section in white indicates no corrosion (X). No corrosion was observed in STS304 under any conditions with an intensity less than 3 and a duration less than 10 min, and no corrosion was observed in STS316 under any conditions with an intensity below 4 and a duration less than 15 min (except 60 Hz for 15 min). Based on the results above, STS316 is more resistant than STS304 to corrosion after the application of electrical pulses

Corrosion was observed for both types of acupuncture needles, as shown in Fig. 2. For STS304 needles, corrosion was observed after EA was performed at an intensity at least 7 and for a duration of 60 min. The conditions under which corrosion was not observed were duration less than 15 min and intensity less than 3. For STS316 needles, corrosion was observed after performing EA at an intensity greater than 8 and for a duration of 90 min. The conditions under which corrosion was not observed were a duration less than 10 min and an intensity less than 5 (Fig. 3).

### Observation of corrosion of acupuncture needles after EA stimulation

**Fig. 4:**
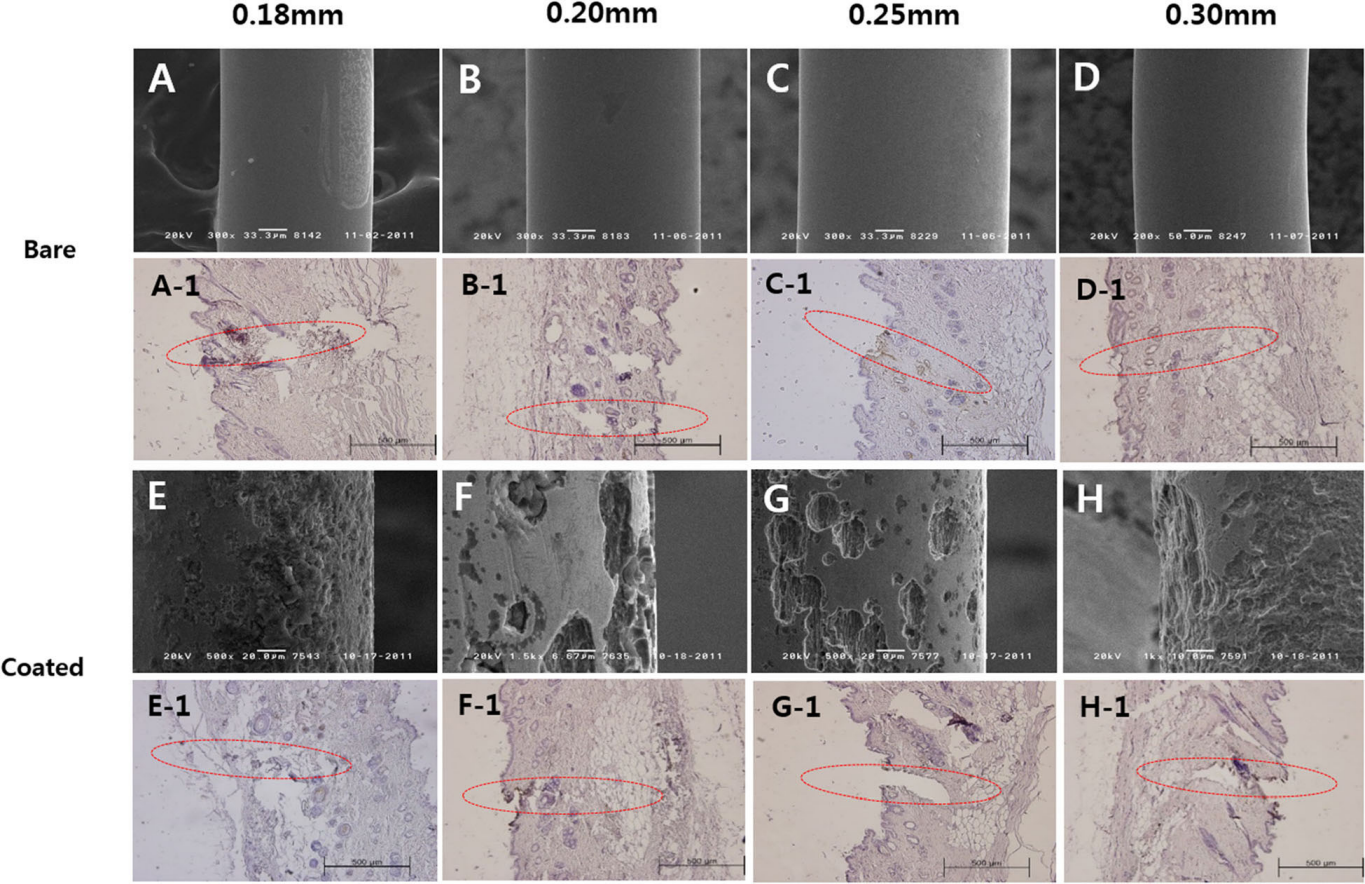
Electron micrographs of STS316 needles after applying EA to tissues, and TUNEL assay results. **a**, **b**, **c**, **d**: Non-coated STS316 needle, **e**, **f**, **g**, **h**: Coated STS316 needles, and dashed figure is TUNEL assay correponding with its panel label.  After applying electricity, corrosion was not observed in non-coated STS316 needles, as shown by electron microscopy. In coated STS316 needles, corrosion was observed such that parts of the needle had fallen apart and grooves had formed. The TUNEL assay results showed suspected cell necrosis (brown-colored TUNEL-positive areas) in areas where non-coated 0.25-mm STS316 needles (C-1), coated 0.2-mm STS316 needles (F-1), and coated 0.3-mm STS316 needles (H-1) had been inserted. The needle insertion sites are circled

Electron microscopy showed that corrosion was not observed for non-coated STS316 needles of any thickness after electrical stimulation, while corrosion, which manifested as peeling or grooves in the surface, was detected for all thicknesses of coated needles (Fig. 4).

### Histological observation of EA-treated areas

**Fig. 5:**
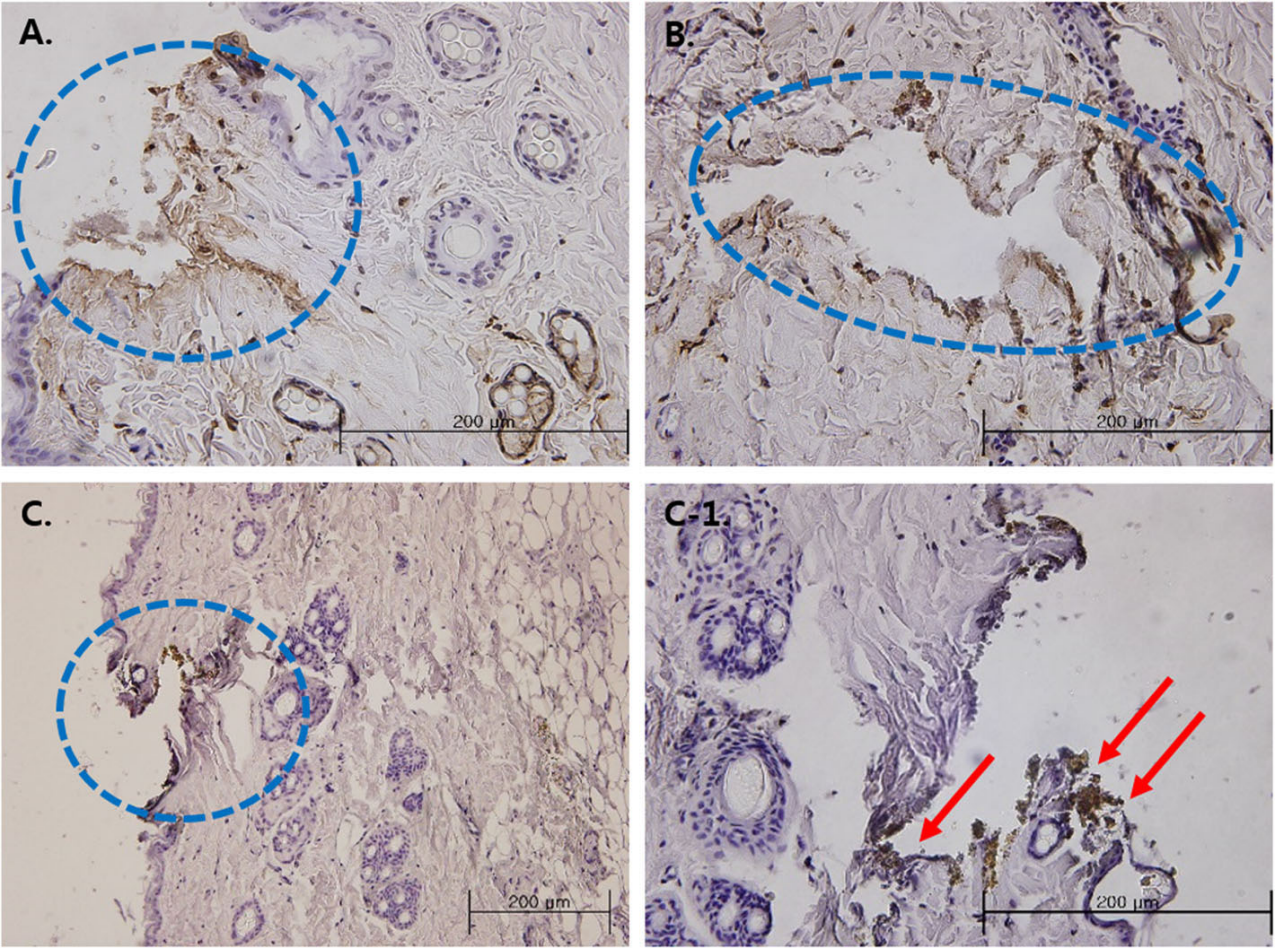
Cell necrosis and corrosion byproducts in tissues. **a**: Non-coated 0.25-mm STS316 needle, **b**: Coated 0.3-mm STS316 needle, **c**: Coated 0.2-mm STS316 needle, **C-1**: Coated 0.2-mm STS316 needle. Cell necrosis was suspected (circled) around the insertion site. Note, however, that actual cell necrosis was detected with non-coated 0.25-mm STS316 needles and coated 0.3-mm STS316 needles, but needle corrosion byproducts were only detected with coated 0.2-mm STS316 needles (arrows)

The TUNEL assay results showed necrosis in the areas where the non-coated 0.25-mm EA needles or the coated 0.3-mm needed had been inserted (Table 3, Figs. 4 and 5). Necrosis was not observed in the areas around the 0.2-mm coated STS316 needle insertion sites, but corrosion byproducts were observed (Fig. 5, arrows).

**Table 3:** Corrosion of STS316 after EA application in tissue and TUNEL assay results (NPN: Number of pixels with tissue necrosis, NPT: Number of pixels with total tissue)

		0.18 mm	0.20 mm	0.25 mm	0.30 mm
Uncoated	Needle	X	X	X	X
	TUNEL	–	–	+	–
	(NPN/NPT)	35/66743	22/62854	1437/56464	28/97992
Coated	Needle	O	O	O	O
	TUNEL	–	±	–	+
	(NPN/NPT)	56/54079	1196/86717	62/65122	1381/67015

(X No corrosion, O Corrosion, TUNEL negative, +: TUNEL positive, ±: suspected TUNEL positive)

### Cytotoxicity evaluation

**Fig. 6:**
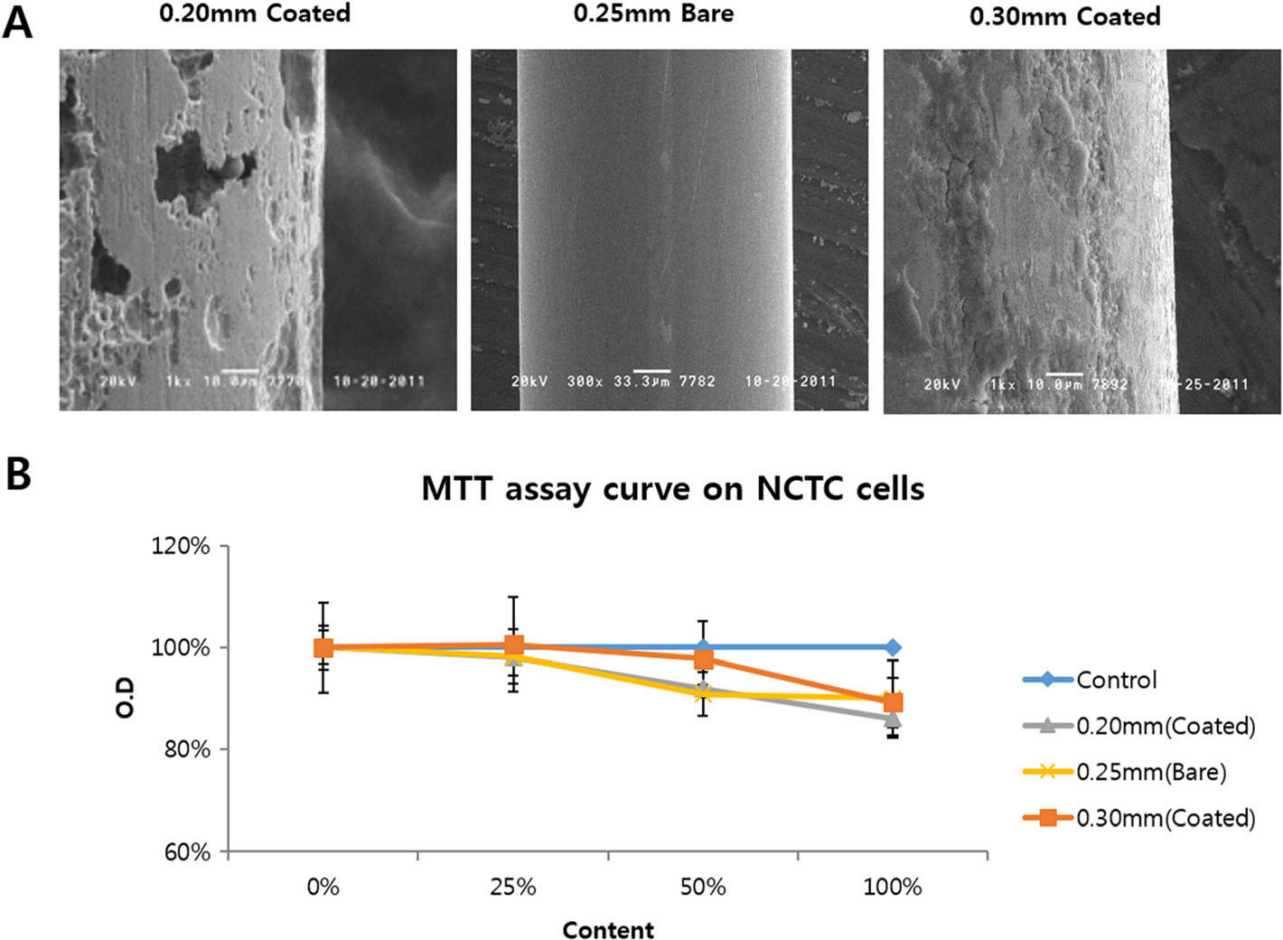
Cytotoxicity of EA byproducts in NCTC cells. **a**: Needles used in the cytotoxicity experiments. Corrosion was not observed in non-coated 0.25-mm STS316 needles but was found in coated 0.2- and 0.3-mm STS316 needles. **b**: MTT assay in NCTC cells. Although the difference was not statistically significant, approximately 10% cytotoxicity was observed 100% media that had been subjected to electrical stimulation with the needle was applied to the cells

The cytotoxicity experiment, which used MTT assays, showed that cell viability tended to decrease as the amount of the media to which electrical stimulation had been applied with the needle increased. When the pure media without electricity application was considered as a control, 10% cytotoxicity was observed for the needle with 100% media with electrical application. Corrosion was not observed in the non-coated 0.25-mm STS316 needle but was detected in the coated 0.2- and 0.3-mm STS316 needles (Fig. 6).

## Discussion

Stainless steel (STS) is a steel alloy with at least 11% chromium content by mass. Chromium oxides can prevent surface corrosion, but not completely under all conditions; however, much less rust and corrosion do occur than in ordinary steel [9]. Furthermore, if a needle is corroded by the application of electricity, the human body can be exposed to harmful substances, such as nickel ions.

Material safety based on the intensity and duration of electrical stimulation was assessed using STS304 and newly developed STS316 needles (0.18, 0.2, 0.25, and 0.3 mm thicknesses). The results showed corrosion in coated STS304 needles of all thicknesses at intensities over 6 or more than 60 min of application, and corrosion was observed on the STS316-coated needles with electricity intensities greater than 8 or applications of 90 min. The high corrosion resistance of coated STS316 needles compared with coated STS304 needles is believed to be attributable to the reduced Cr content and increased Ni content, which improves corrosion resistance, and the addition of molybdenum, which increases creep resistance, in the STS316 needles [10–12].

Since the coated STS316 needles showed stronger corrosion resistance after EA application than the coated STS304 needles, tissue damage due to EA application was examined using coated or non-coated STS316 needles. In the in vivo corrosion tests, corrosion was observed on all coated needles but not on uncoated needles. This result is consistent with the results of Kwon et al. [13], who reported that the presence of a silicone coating, not the needle material, greatly affected the degree of corrosion when electrical stimulation was applied.

While corrosion was observed on all of the coated needles, cell necrosis in the tissues treated with EA using non-coated 0.25-mm STS316 needles and coated 0.3-mm STS316 needles was observed via TUNEL assay. Cell necrosis was suspected in tissues that underwent electrical stimulation using coated 0.2-mm STS316 needles, and corrosion byproducts were also observed (Fig. 4). The non-coated 0.25-mm STS316 needles showed no corrosion but did lead to cell necrosis. The coated STS316 needles showed corrosion at all thicknesses, yet necrosis was suspected or observed only with the 0.2- and 0.3-mm needles. Assessing the presence of corrosion and of cell necrosis after applying electricity showed that corrosion did not always lead to cell necrosis. Shalahinejad et al. found that there is no direct correlation between cell viability and corrosion resistance [14]. Result from previous studies also support the results of this study.

Slight cytotoxicity was observed for all the needles, but the difference was not significant. In particular, in the case of the non-coated 0.25-mm STS316 needles, in which corrosion was not observed, 10% cytotoxicity was detected. These results suggest that cytotoxicity can occur even in the absence of the corrosion of the needle.

Recently, Xie, et al. [15] reported the harmful effects of electroacupuncture depending on the quality of needles used in clinical practices and emphasized the importance of quality control procedures in needle manufacturing rather than the risk of electroacupuncture stimulation. Zhang, et al. [16] confirmed the low risk of corrosion under the condition of electric stimulation used in actual clinical practices using STS 304 needles and suggested the safety of its clinical use. Our study results showed low harmful effects on the human body associated with corrosive materials from electroacupuncture that can be developed during stronger electric stimulation. Thus, the results above suggest that the risk of electroacupuncture operation currently used in clinical practices is low.

This study results showed no direct relationship between corrosion byproducts after electrical stimulation and cell necrosis. There may be three possible reasons for cell necrosis to occur during EA stimulation. First, cell necrosis by heat is a possibility. When electricity is applied to the needle, heat is produced due to the resistance of the human body and the friction of muscle movements, which can cause tissue necrosis and tissue damage. Second, cell necrosis by reactive oxygen species (ROS) could occur. ROS are unstable oxygen species that can damage cell membranes, DNA, and other cell structures, and depending on the extent of the damage, cell functions can be lost [17]. According to a previous study, a nanosecond pulsed electric field may increase the ROS level, which can cause cell damage [18]. In our study, tissue damage could also be predicted to occur due to ROS production by electrical stimulation. Third, electrical shock could occur. When the needle is inserted into the tissue, there may be a small space between the tissues and the needle; after EA application, electrical charge accumulates in the space and can be discharged, generating sparks in the space and causing tissue necrosis by electrical shock.

Based on the results above, it is appropriate to use uncoated (bare) needles composed of STS316 material for EA treatment in a clinical setting. In addition, corrosion byproducts produced by EA corrosion are not directly associated with cell necrosis; slight cytotoxicity and cell necrosis can develop in the process of applying the electricity. Because the relationship between corrosion byproducts and cell necrosis was examined by applying strong electrical stimulation with STS316, which is relatively stable, accurate criteria for the point at which cell necrosis develops are not suggested. Additional experiments are necessary to accurately identify the cause of cell necrosis. The safety of EA stimulation and accurate criteria for such stimulation as determined by additional studies can be used as the basis for clinical applications in the future.

## Data Availability

The datasets generated during and/or analyzed during the current study are available from the corresponding author on reasonable request.

## Abbreviations

EA: Electroacupuncture
ROS: Reactive oxygen species
STS: Stainless steel
TUNEL: Terminal deoxynucleotidyltransferase-mediated dUTP nick end labeling
